# Efficacy and safety of polymyxin E sulfate in the treatment of critically ill patients with carbapenem-resistant organism infections

**DOI:** 10.3389/fmed.2022.1067548

**Published:** 2022-12-29

**Authors:** Xin Lu, Cejun Zhong, Yanbin Liu, Hui Ye, Junyan Qu, Zhiyong Zong, Xiaoju Lv

**Affiliations:** Center of Infectious Diseases, West China Hospital, Sichuan University, Chengdu, China

**Keywords:** polymyxin E sulfate, carbapenem-resistant organisms, efficacy, nephrotoxicity, acute kidney injury

## Abstract

**Objective:**

Polymyxins are currently the last line of defense in the treatment of carbapenem-resistant organisms (CRO). As a kind of polymyxin available for clinical use in China, we aim to explore the efficacy and safety of colistin sulfate (Polymyxin E sulfate, PES) in this study.

**Methods:**

This real-world retrospective study included 119 patients diagnosed with CRO infection and treated with PES for more than 72 h, from May 2020 to July 2022 at West China Hospital. The primary outcome was clinical efficacy at the end of treatment, and secondary outcomes included microbial response, in-hospital mortality and incidence of nephrotoxicity.

**Results:**

The effective clinical and microbiological responses were 53.8% and 49.1%, respectively. And the in-hospital mortality was 27.7%. Only 9.2% of patients occurred with PES-related nephrotoxicity. Multivariate analysis revealed that duration of PES was an independent predictor of effective therapy, while age-adjusted Charlson comorbidity index (aCCI) and post-treatment PCT(p-PCT) were independent risk factors for poor outcome.

**Conclusions:**

PES can be a salvage treatment for CRO-induced infections with favorable efficacy and low nephrotoxicity. The treatment duration of PES, aCCI and p-PCT were factors related to the clinical effectiveness of PES.

## Introduction

On a global scale, drug-resistant bacteria have been associated with a substantial amount of death and morbidity, posing a substantial risk to public health. Among them, multi-drug resistant gram-negative bacteria (MDR-GNB), especially carbapenem-resistant *Acinetobacter baumannii* (CRAB), and carbapenem-resistant *Pseudomonas aeruginosa* (CRPA) and carbapenem-resistant Enterobacteriaceae (CRE) have been classified as a critical threat by the WHO (1). However, with the slow development of new antimicrobials, clinicians are progressively stuck without drugs and searching for alternatives that can alleviate the current dire situation. As a result, polymyxins have been experiencing a renaissance as a last-line defense in the current treatment of infections caused by carbapenem-resistant organisms (CRO).

Polymyxins are antimicrobial peptides produced by *Bacillus polymyxa* and used clinically as polymyxin B and colistin (polymyxin E), which were first approved in the 1950s. However, its toxicity and the availability of other safer and more effective antibacterial drugs contributed to a significant decline in its use since the 1970s (2). With the current rapid development of MDR-GNB and the improvement of the technology of polymyxin preparation, this “old” antibiotic has been reintroduced. At present, there are three types of polymyxins for injection available in domestic and international markets, including colistimethate sodium (CMS), polymyxin B sulfate (PBS) and colistin sulfate (Polymyxin E sulfate, PES) (3). Of these, CMS is an unactivated pre-drug that needs to be transformed into active polymyxin *in vivo* to exert bactericidal effects, almost 60-70% of which is excreted through the kidneys (4). The latter two drugs do not require conversion and can act directly, mainly eliminated by non-renal routes (5).

Polymyxin is a concentration-dependent antibiotic (6) whose most prevalent side effects include nephrotoxicity and neurotoxicity. There are numerous clinical trials conducted abroad on CMS and PBS. It has not been identified which medication is preferable for achieving greater efficacy and a lower rate of adverse effects when used to treat infections caused by CRO. As the earliest medicine to be launched internationally, CMS has received the most attention. An initial study (7) demonstrated that CMS was less nephrotoxic than PBS. In order for CMS to exert its bactericidal effect, it must be converted to polymyxin *in vivo* (8), which delays the commencement of its activity, while it is imperative to treat people with serious infections promptly. Therefore, clinicians prefer to use PBS without conversion. Even more recently, many studies have shown that the probability of acute kidney injury (AKI) is less with PBS than that with CMS (9, 10). Actually, both nephrotoxicity and other side effects are temporary and reversible upon dosage decrease or medication termination. However, Lin et al. (11) demonstrated that nebulized PBS is hazardous to alveolar epithelial cells, and another case (12) showed that intranasal PBS can result in respiratory arrest. In addition, the most recent Sanford Antimicrobial Therapy Guidelines stipulate (13) that PBS should not be utilized for nebulized therapy. In China, PES, which is the polymyxin E but not pre-drug as CMS, has been marketed in 2018. Similar metabolic routes exist between PES and PBS, and both can operate directly without conversion. Perhaps PES can complement each other to obtain considerable efficacy and less incidence of adverse effects. There are few clinical reports of PES, so we conducted a real-world research retrospectively on efficacy and safety of PES used in patients with CRO infections aimed to give a reference for the clinical use of PES.

## Materials and methods

### Study design

The study was performed in a 4300-bed tertiary teaching general hospital, the West China Hospital of Sichuan University, Chengdu, in southwest China. The Ethics Committee on Biomedical Research, West China Hospital of Sichuan University, approved this retrospective study with exemption from informed consent. The diagnosis of CRO infection was made by two clinicians based on culture of CRO from sterile or eligible specimen species and high suspicion of CRO infection with respect to a combination of the patient’s clinical symptoms, signs and examination findings, such as fever pattern and inflammatory indicators, despite the absence of positive pathogenic bacterial culture results. Inclusion criteria consisted of age ≥18 years, use of PES (Marketed in the mainland on April 16, 2018 by Shanghai SPH New Asia Pharmaceutical Co., Ltd., Shanghai, China) for ≥72 h, and a diagnosis of infection caused by CRO. The patients aged less than 18 years, on medication for less than 72 h, with pregnancy and incomplete clinical data were excluded.

The patient data was collected from the electronic medical record system of hospital. It mainly includes basic demographic characteristics, Acute Physiology and Chronic Health Evaluation (APACHE) II score, Sequential Organ Failure Assessment (SOFA) score, the aged-adjusted Charlson comorbidity index (aCCI) (14), length of hospitalization, biochemical parameters, infection indices, underlying diseases, complications, invasive procedures, infection sites and causative agents, exposure to antimicrobial therapies, duration of treatment, simultaneous coinfections and use of other antibiotics, concomitant nephrotoxic agents, clinical and microbiological responses, safety evaluation, in-hospital mortality, etc.

According to the domestic consensus (15), the current recommended intravenous dose of PES is 1 million U–1.5 million U/day; the nebulized inhalation dose is 250,000–500,000 U/day; and the recommended dose of intrathecal injection is not stated. Prescription and duration of PES therapy were determined by the attending physician based on the clinical condition of individual patients under the suggestion of consensus. The primary outcome endpoint was the assessment of clinical efficacy at the time of drug discontinuation. Secondary outcome endpoints were microbial response, in-hospital mortality and incidence of adverse drug reaction.

### Definition

The clinical efficacy of PES therapy was evaluated by clinical and microbiological criteria at the time of withdrawal of the drug. Only the first course of treatment was analyzed for patients who received more than one treatment with PES. By comparing the patient’s baseline infection status with those after therapy, complete or partial alleviation of symptoms and signs were defined as favorable clinical responses. The persistence or aggravation of symptoms and indications was categorized as an unfavorable clinical response. Using matrix-assisted laser desorption/ionization time-of-flight mass spectrometry (MALDI-TOF/MS), the pathogenic bacteria were extracted and identified. The antimicrobial susceptibility was determined in accordance with experts consensus (16) referring to the epidemiological cutoff (ECOFF) values for polymyxins and the pharmacokinetics/pharmacodynamics (PK/PD) cutoff values, taking into account of clinical efficacy analysis of polymyxins and the current cutoffs values of European Committee on Antimicrobial Susceptibility Testing (EUCAST) (17)and United States Committee on Antimicrobial Susceptibility Testing (USCAST) (18). Empirically administered patients without positive cultural results were excluded on assessment for microbiological response. Bacteriological eradication was identified as two consecutive negative culture findings of the infection site specimen following drug administration. Bacteriological clearance and reduction in bacterial load were considered as valid response. The absence of the above microbiological responses or continuous surveillance of the same causative agent was deemed invalid.

Nephrotoxicity is the most frequent adverse effect of polymyxin. As to evaluating drug-related nephrotoxicity, patients with CKD or baseline estimated glomerular filtration rate (eGFR) < 60 ml/min per 1.73 m^2^ were excluded, as well as patients who had undergone continuous renal replacement therapy (CRRT) before dosing or on CRRT without nephrotoxicity during drug administration. The definition of AKI was a rise in serum creatinine (SCr) by ≥ 0.3 mg/dl within 2 days, a 50% increase in SCr over baseline within 7 days or urine output < 0.5 ml/kg/hour for 6 hours, according to the Kidney Disease Improving Global Outcomes (KDIGO) criteria (19). Baseline SCr and eGFR were measured as the most recent figures available at the initiation of the treatment with PES.

### Statistical analysis

The data was conducted through the statistical data analysis software IBM SPSS Statistics 26.0 (IBM Corp. in Armonk, NY, USA). For continuous variables, the normally distributed data was described by mean and standard deviation (SD), and statistical inference was made by independent *t*-test. While non-normal ones were described by median and interquartile range (IQR) and inferred by Mann–Whitney U-test. Categorical variables were analyzed using the chi-square test or Fisher’s exact probability method. Wilcoxon signed rank test was performed to analyze rank variables. All variables were divided into two groups for comparison based on clinical response outcomes. In the univariate analysis, variables with *P* values < 0.1 were enrolled in the multivariate logistic regression model for analysis. Two-tailed *P* values less than 0.05 indicate a significant difference.

## Results

**FIGURE 1:**
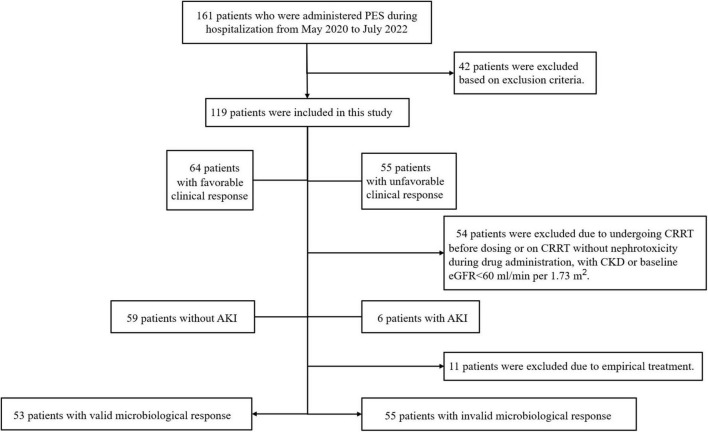
Study grouping and flow chart. PES, polymyxin E sulfate; CRRT, continuous renal replacement therapy; CKD, chronic kidney disease; AKI, acute kidney injury.

According to data from the hospital’s electronic medical record information system, there were 161 patients who were administered PES during hospitalization from May 2020 to July 2022. A total of 119 patients were enrolled based on inclusion and exclusion criteria (Figure 1). Table 1 showed the key demographic and clinical characteristics of these patients to describe the general situation and compare the deferent clinical responses. The mean age of all patients was 59.62 ± 15.07 years old, including 83(69.7%) males, with an average BMI being 23.44(IQR 21.06–26.17) kg/cm^2^. Their median length of hospitalization was 35(23–59) days. The most common underlying disease was cardiovascular disease (65.5%), followed by diabetes (29.4%) and chronic pulmonary disease (26.9%). Eighty-six patients (72.3%) were complicated with sepsis, and the second highest rate of complications were respiratory failure (68.9%) and hepatic insufficiency (68.9%). Almost all received invasive assisted ventilation, accounting for 92.4%, accompanied by a high rate of vasoactive agents (93.3%).

**TABLE 1:** Demographics and clinical characteristics of 119 patients receiving polymyxin E sulfate.

Variables	Total	Favorable clinical response (*n* = 64, 53.8%)	Unfavorable clinical response (*n* = 55, 46.2%)	*P*-value
Age, years, mean ± SD	59.62 ± 15.07	57.48 ± 16.36	62.12 ± 13.12	0.094
BMI, kg/m^2^, median (range)	23.44(21.06–26.17)	24.50 ± 4.48	22.92 ± 3.52	0.037
Sex, male (%)	83(69.7%)	43(67.2%)	40(72.7%)	0.512
APACHE II score, mean ± SD	19.97 ± 7.45	20.78 ± 7.86	19.02 ± 6.90	0.200
SOFA score, mean ± SD	9.92 ± 3.92	9.50(6.00–12.00)	10.00(7.00–14.00)	0.101
aCCI, median (range)	6.00(4.00–8.00)	5.52 ± 2.45	6.60 ± 2.86	0.028
Days of hospitalization, days, median (range)	35.00(23.00–59.00)	41.50(26.50–70.50)	29.00(18.00–45.00)	0.012
**Underlying diseases (%)**				
Diabetes	35(29.4%)	17(26.6%)	18(32.7%)	0.462
Cardiovascular disease	78(65.5%)	41(64.1%)	37(67.3%)	0.713
Cerebrovascular disease	23(19.3%)	12(18.8%)	11(20.0%)	0.863
Chronic liver disease	20(16.8%)	7(10.9%)	13(23.6%)	0.065
Acute severe pancreatitis	19(16%)	11(17.2%)	8(14.5%)	0.695
Chronic pulmonary disease	32(26.9%)	16(29.1%)	16(25%)	0.616
CKD	13(10.9%)	9(14.1%)	4(7.3%)	0.236
Solid-organ tumor	20(16.8%)	11(20%)	9(14.1%)	0.388
Hematological tumor	9(7.6%)	1(1.6%)	8(14.5%)	0.020
Organ transplantation	11(9.2%)	4(6.3%)	7(12.7%)	0.224
**Complications (%)**				
Sepsis	86(72.3%)	41(64.1%)	45(81.8%)	0.031
Respiratory failure	82(68.9%)	47(73.4%)	35(63.6%)	0.249
Hepatic insufficiency	82(68.9%)	40(62.5%)	42(76.4%)	0.103
Gastrointestinal hemorrhage	71(59.7%)	38(59.4%)	33(60.0%)	0.945
Thrombocytopenia	43(36.1%)	20(31.3%)	23(41.8%)	0.232
Coagulation disorders	61(51.3%)	29(45.3%)	32(58.2%)	0.161
**Invasive procedures (%)**				
Assisted invasive ventilation	109(92.4%)	62(96.9%)	48(87.3%)	0.104
CRRT	50(42.0%)	25(39.1%)	25(45.5%)	0.481
General anesthetic surgery	54(45.4%)	32(50%)	22(40.0%)	0.275
**Other medication (%)**				
Vasoactive agents	111(93.3%)	61(95.3%)	50(90.9%)	0.556
Steroids	104(87.4%)	52(81.3%)	52(94.5%)	0.029

SD, standard deviation; APACHE, Acute Physiology and Chronic Health Evaluation; SOFA, Sequential Organ Failure Assessment; BMI, body mass index; aCCI, aged-adjusted Charlson comorbidity index; CKD, chronic kidney disease; CRRT, continuous renal replacement therapy.

Among all patients diagnosed with CRO infections, the pulmonary infections was 83.2%, followed by bloodstream infections (25.2%), abdominal infections (13.4%), urinary tract infections (10.1%), and intracranial infections (5.0%). Among them, concurrent CRO infections in multiple sites were 37.8%. CRAB (72.3%) was the most frequently monitored CRO in sterile specimens, followed by carbapenem-resistant *Klebsiella pneumoniae* (CRKP) (33.6%), *Stenotrophomonas maltophilia* (20.2%), CRPA (9.2%). Among them, 40.3% of patients were infected with two or more CROs simultaneously (Table 2). The MIC value ≤ 2mg/L tested by broth microdilution was defined as sensitive and ≥4mg/L as resistant according to the drug sensitivity determination criteria mentioned above. All isolated CROs in this study were sensitive to PES.

**TABLE 2:** The clinical characteristics of CRO infections in 119 patients receiving polymyxin E sulfate based on different clinical responses.

Variables	Total	Favorable clinical response (*n* = 64, 53.8%)	Unfavorable clinical response (*n* = 55, 46.2%)	*P*-value
**Infection site (%)**				
Pulmonary infections	99(83.2%)	56(87.5%)	43(78.2%)	0.175
Bloodstream infections	30(25.2%)	16(25%)	14(25.5%)	0.955
Abdominal infection	16(13.4%)	7(10.9%)	9(16.4%)	0.387
Urinary tract infections	12(10.1%)	9(14.1%)	3(5.5%)	0.120
Intracranial infections	6(5.0%)	5(7.8%)	1(1.8%)	0.285
≥2 Infection Sites	45(37.8%)	25(39.1%)	20(36.4%)	0.762
**CRO (%)**				
CRAB	86(72.3%)	51(79.7%)	38(61.9%)	0.124
CRKP	40(33.6%)	23(35.9%)	18(32.7%)	0.850
CRPA	11(9.2%)	4(6.3%)	7(12.7%)	0.224
Other CREs	5(4.2%)	1(1.6%)	4(7.3%)	0.276
SM	24(20.2%)	13(20.3%)	11(20.0%)	0.966
≥ 2 Types of CRO	48(40.3%)	24(37.5%)	24(43.6%)	0.496
**Baseline condition**				
Serum albumin, g/L, mean ± SD	32.83 ± 5.09	33.55 ± 4.30	31.99 ± 5.81	0.095
eGFR, mL/min/1.73 m^2^, mean ± SD	79.91 ± 36.68	84.24 ± 37.36	74.86 ± 35.55	0.165
SCr, μmol/L, median (range)	78.00(55.00–129.00)	75(52.75–110.75)	79(57–147)	0.309
WBC, 10^9^/L, median (range)	10.05(7.25–14.58)	10.88(8.29–16.02)	9.1(5.84–13.16)	0.050
Neutrophil count, 10^9^/L, median (range)	8.48(0–13.63)	8.94(6.93–14.37)	7.77(4.54–11.86)	0.059
PCT, μg/L, median (range)	1.62(0.41–5.59)	1.32(0.40–5.62)	1.85(0.43–5.59)	0.583
**Post-treatment indicators**				
p-Neutrophil count, 10^9^/L, median (range)	8.43(4.78–12.75)	8.62(5.58–12.55)	7.8(4.23–13.27)	0.304
p-PCT, μg/L, median (range)	1.16(0.30–3.83)	0.46(0.18–1.65)	2.76(0.98–9.97)	0.000

SD, standard deviation; CRO, carbapenem-resistant organisms; CRAB, carbapenem-resistant *Acinetobacter baumannii*; CRKP, carbapenem-resistant *Klebsiella pneumoniae*; CRPA, carbapenem-resistant *Pseudomonas aeruginosa*; CRE, carbapenem-resistant Enterobacteriaceae; SM, *Stenotrophomonas maltophilia*; eGFR, estimated glomerular filtration rate; SCr, serum creatinine; WBC, white blood cell count; PCT, procalcitonin; p-, post-treatment.

In terms of antimicrobial therapy, PES was administered to 25 patients (21%) within 24 h of CRO reported. After several antimicrobial medication failures, such as meropenem or tigecycline, 37 patients (31.1%) were treated with PES before the CRO culture results available. The majority of patients experienced intravenous systemic therapy, while three patients were treated solely with PES nebulization. A combination of topical PES was administered to 40.3% of patients via aerosolization and 5% via intracerebroventricular injection. Only 39 patients (32.8%) were treated with loading dosage, and 13 (10.9%) of them received a dose that was twice as high as the maintenance dose. The great majority of patients in our study accepted the consensus-recommended intravenous and nebulized dosages, as well as the daily intrathecal injection dose of 50,000 U-100,000 U/d. With a maximum daily dose of 2 million U intravenously and 1.5 million U nebulized, only a very tiny minority of patients had an overdose, and no AKI was observed (not shown in Table 3). PES monotherapy for CRO infections was used in only 8 patients (6.7%). Combination therapy included with tigecycline, carbapenems,β-lactamase inhibitor conjugates, aminoglycosides, quinolones, fosfomycin, aztreonam, etc. The therapeutic duration of PES was 11(7–16) days, and the cumulative intravenous dose was 13.50(9–20) MU. Overall, the rates of favorable clinical response and effective microbiological response were 53.8% and 49.1% respectively. The rate of in-hospital mortality was 27.7% (Table 3).

**TABLE 3:** The treatment methods of polymyxin E sulfate and outcomes based on different clinical responses.

Variables	Total	Favorable clinical response (*n* = 64, 53.8%)	Unfavorable clinical response (*n* = 55, 46.2%)	*P*-value
**Timing of medication^a^ (%)**				
>24 h post culture positive	57(47.9%)	29(45.3%)	28(50.9%)	0.829
<24 h post culture positive	25(21%)	14(21.9%)	11(20.0%)	
Before CRO reported	37(31.1%)	21(32.8%)	16(29.1%)	
**Administration method (%)**				
IV	62(52.1%)	29(45.3%)	33(60.0%)	0.119
IH	3(2.5%)	3(4.7%)	0(0.0%)	
IV + IH	48(40.3%)	27(42.2%)	21(38.2%)	
IV + IVT	6(5.0%)	5(7.8%)	1(1.8%)	
**First dose administration (%)**				
None^b^	80(67.2%)	49(76.6%)	31(56.4%)	0.065
Addition^c^	26(21.8%)	10(15.6%)	16(29.1%)	
Doubled^d^	13(10.9%)	5(7.8%)	8(14.5%)	
**Treatment course, median (range)**	11(7–16)	13(10–17.75)	9(5–14)	0.000
≤7 days (%)	32(26.9%)	8(12.5%)	24(43.6%)	0.000
8–14 days (%)	54(45.4%)	33(51.6%)	21(38.2%)	
>14 days (%)	33(27.7%)	23(35.9%)	10(18.2%)	
Daily dose of IV, median (range)	150(150–150)	150(150–150)	150(150–150)	0.717
Cumulative dose of IV, median (range)	1350(900–2000)	1700(1056.25–2250)	950(525–1825)	0.000
**Types of co-administration^e^ (%)**				
Monotherapy combination therapy	8(6.7%)	7(10.9%)	1(1.8%)	0.979
1 type of other agents	63(52.9%)	30(46.9%)	33(60%)	
2 types of other agents	41(34.5%)	22(34.4%)	19(34.5%)	
≥3 types of other agents	7(5.9%)	5(7.8%)	2(3.6%)	
**Outcome (%)**				
In-hospital mortality	33(27.7%)	12(18.8%)	21(38.2%)	0.018
Patients occurred with AKI^f^	6(9.2%)	3(7.7%)	3(11.5%)	0.930
Valid microbiological response^g^	53(49.1%)	38(62.3%)	15(31.9%)	0.002

CRO, carbapenem-resistant organisms; AKI, acute kidney injury; IV, intravenously guttae; IH, inhalation; IVT, intraventricular injection.

^a^Timing of colistin sulfate dosing before and after the identification of CRO. 37 patients were administrated with colistin sulfate before CRO reported, and only 11 patients were lack of positive bacterial culture results.

^b^The first dose is not a loading dose.

^c^The loading dose is less than two times the maintenance dose.

^d^The loading dose is two times the maintenance dose.

^e^Other antimicrobial drugs used in conjunction with PES therapy, include tigecycline, carbapenems, β-lactamase inhibitor conjugates, aminoglycosides, quinolones, *fosfomycin*, aztreonam, etc.

^f^Fifty-four patients were excluded due to undergoing continuous renal replacement therapy (CRRT) before dosing or on CRRT without nephrotoxicity during drug administration, with CKD or baseline eGFR < 60 ml/min per 1.73 m^2^.

^g^Eleven patients were excluded due to empirical therapy without positive bacterial culture results.

There was no difference between the clinical response groups in age, gender, APACHE II score, SOFA score, invasive procedures, pathogenic bacteria, infection site of CRO, the timing and method of PES administration, the first dose administration status, types of co-medication, baseline renal function and incidence of AKI. In comparison to unfavorable clinical responses, patients in the favorable clinical response group had higher BMI and lower aCCI, longer hospital stays and treatment courses, less underlying disease with hematologic tumor and steroid use, less complicated with sepsis, higher cumulative intravenous dose, lower post-treatment PCT (p-PCT) values, less in-hospital mortality, and higher rates of effective microbial response. In multifactorial analysis, there were substantial differences in aCCI [*P* = 0.028; OR = 1.208 (95%CI 1.021–1.430)]and p-PCT [*P* = 0.002; OR = 1.219 (95%CI 1.077–1.381)] across clinical response groups. Compared to those treated for 7 days and less, there were significant differences in patients with 8–14 days of treatment [*P* = 0.013; OR = 0.242 (95%CI 0.079–0.746)] and in patients with more than 14 days of treatment [*P* = 0.026; OR = 0.249 (95%CI 0.073–0.847)] (Table 4).

**TABLE 4:** Multivariate logistic regression of clinical response of polymyxin E sulfate treatment.

Variables	*P*-value	OR (95%CI)
aCCI	0.028	1.208(1.021–1.43)
**Treatment course**		
8–14 days	0.013	0.242(0.079–0.746)
>14 days	0.026	0.249(0.073–0.847)
Hematological tumor	0.081	7.437(0.781–70.816)
p-PCT	0.002	1.219(1.077–1.381)

aCCI, aged-adjusted Charlson comorbidity index; p-PCT, post-treatment procalcitonin.

To minimize the impact of poor underlying renal function and patients on CRRT on AKI estimation, 65 patients were included for evaluation of PES-related AKI. Among them, 6 patients (9.2%) developed AKI (Table 3). Three of the six patients with AKI had acute severe pancreatitis and experienced non-AKI CRRT after the onset of nephrotoxicity. Two of them underwent surgery after 14 days of PES treatment. Six individuals developed AKI in the time range of 4–9 days after drug administration, three patients experienced a steady decrease in creatinine after discontinuing PES, and one patient died the day after developing AKI due to septic shock (not shown in Table 3). No other adverse reactions such as neurotoxicity and skin pigmentation were observed in all patients.

## Discussion

In our study, severe pneumonia dominated since the majority of patients experienced respiratory failure or required mechanical ventilation. All patients had severe underlying illness and greatly compromised immune function, frequently with several CRO infections, with CRAB and CRKP being the most prevalent causal agents, separately. The current resistance rates of CRAB, CRKP, and CRPA to PES, as determined by the China Antimicrobial Surveillance Network (CHINET) in the first half of 2022, are 0.8, 7.2, and 4.4%, respectively. As the resistance rate of these gram-negative bacilli to carbapenems increases year by year, PES as a salvage therapy for infections shown favorable effectiveness and a low incidence of AKI in this trial. Comparing groups by efficacy, we discovered that duration of PES was predictors of effective therapy, while aCCI and high p-PCT was independent risk factors for poor efficacy.

Due to the exclusive clinical utilization of PES in China, few studies have been reported to evaluate its efficacy and safety, and even fewer PK/PD researches. Numerous therapeutic applications have been extrapolated from PBS investigations because of the similar structure and mode of metabolism of both substances. According to consensus (15), loading doses are recommended for both PBS and CMS. Among them, PBS needs to be administered according to body weight, while CMS needs to adjust the dose depending on renal function. In our study, PES was not treated with weight-based dosing and the dose was not adjusted based on renal function. In two recently reported clinical PK/PD investigations on PES (20, 21), creatinine clearance (CrCL) was a covariate of PES clearance, which is conflicting with the conclusion that PES was independent with CrCL as reported by Peng et al. (22) and the earlier studies (4, 23) demonstrating that PBS clearance was not well associated by CrCL. The dose required to achieve the target therapeutic concentration varies among patients with different levels of renal function. Therefore, the current consensus (15) recommended dose is only indicated for patients with pathogenic bacteria-induced infections with MIC ≤ 0.5 μg/ml and renal insufficiency (CrCL ≤ 50 ml/min) (21). However, the pharmaco-kinetic models used in the aforementioned investigations with minimal sample sizes were limited to intravenous administration, ignoring the effects produced by nebulization. There may be more complex relationship between CrCL and PES clearance, and substantial samples of clinical PK/PD studies are still required to guide PES treatment.

Compared to earlier or retrospective clinical investigations on PES with smaller sample sizes, the treatment efficacy ranged between 73% and 89% (24–28). In contrast, the good clinical response rate in our trial was 53.8%, comparable to the 59.52% reported by Yu et al. (20). Numerous clinical efficacy rates on CMS and PBS varied from 45% to 88% (29) and 47.3% to 76% (9), respectively. Regarding the parameters impacting the efficacy of the other two medications, the loading dose (30–32), maintenance dose (33, 34), and time of drug administration (35, 36), dosing regimen (33, 37–39), and combination of other agents (40, 41) were the primary areas of concern. In the present investigation, the timing of PES administration, the loading dose, the shape of the loading dose, the mode of administration, and the type of combination were not significantly associated with efficacy based on the suggested maintenance dose. The duration of treatment for PES was strongly associated with effectiveness. Similar to the study of Xia et al. on PBS (42) and consistent with the consensus recommended duration, the treatment groups with durations of 8–14 days and more than 14 days exhibited significantly greater efficacy than those with durations of 7 days or less. Longer treatment duration and greater cumulative doses of medication, while boosting efficacy, can increase the risk of adverse effects in patients, whereas in this trial there was no differences in the prevalence of AKI between efficacy groups. In contrast, prolonged treatment duration results in a longer hospital stay, which raises the financial burden for critically ill patients. Moreover, when considering the poor efficacy of patients with a short course of treatment, it cannot be ruled out that some patients with severely critical situations may pass away during the early stages of drug administration.

Another major discovery was that aCCI had a substantial impact on clinical outcomes. Unlike the APACHE II score and SOFA score, the aCCI, with a higher prediction value than CCI (14), predicts the probability of death from disease based mostly on patients’ comorbidities. In research evaluating the factors that influence the efficacy of polymyxin treatment, there have been no reports of a correlation between aCCI and efficacy. Higher aCCI values suggested that the presence of significant comorbidities and immunocompromised individuals, making it harder to obtain the appropriate clinical response, even with pathogen-sensitive PES. In addition, the other noteworthy finding to emerge from the analysis is that p-PCT was an independent predictor of clinical response. PCT is a reliable biomarker for distinguishing bacterial infections in terms of diagnosis, illness assessment, therapy guidance, and prognosis (43). In the current investigation, p-PCT was considerably lower in the group with a favorable clinical response compared to the group with a unfavorable ones. In the absence of TDM, p-PCT levels were evaluated to assess the response of patients to PES therapy and determine whether to continue treatment. Patients with lower respiratory tract infections and sepsis should terminate antibiotic therapy when p-PCT levels fall below 0.25 and 0.50 ng/ml, respectively (44). As the last line of defense in the treatment of CRO, PES is costly and promotes drug resistance with prolonged administration. Therefore, it is crucial to continuously evaluate PCT together with patient specificity to direct PES in the treatment of infections caused by CRO without TDM.

In our study, the incidence of nephrotoxicity, the most prevalent adverse effect of polymyxin, was 9.2%. In the two cases of CNS infections (45, 46) and one example of pulmonary infection successfully treated by PES (47), no renal damage was reported. In other clinical investigations with smaller samples (20, 24–28), there were a maximum of two incidences of renal injury due to PES. In numerous nephrotoxicity investigations on the other two polymyxins, the incidence of nephrotoxicity at commonly recognized dosages and AKI classifications ranged from 20% to 50% for both medications (48). Compared to these two polymyxins, the incidence of AKI associated with PES was considerably lower. In addition to variances in medications, fundamental patient circumstances, and AKI evaluation criteria (48), the inclusion of varied exclusion criteria for determining nephrotoxicity may contribute to the wide diversity in nephrotoxicity assessments. In this investigation, we omitted the impact of CRRT and baseline renal function since poor baseline renal function may be the consequence of a combination of causes, despite the fact that some literature (49, 50) identified baseline renal impairment as a risk factor for polymyxin-induced nephrotoxicity. However, in our current investigation, the low incidence of PES-related AKI made it difficult to discover relevant factors associated with nephrotoxicity, because of the small sample size. The large-scale, multicenter clinical investigations are still required to figure out the factors driving PES nephrotoxicity.

However, this study has some limitations. Firstly, this was a retrospective, single-center research with a limited sample size and no case controls. Secondly, during the treatment of PES, doctors made judgments about dosage, administration, and duration based on guidelines and personal experience without therapeutic drug monitoring (TDM), making it hard to verify whether appropriate therapeutic concentrations were obtained. Thirdly, because the great majority of patients have invasive assisted breathing with concomitant sedative drugs, it is extremely possible that neurotoxic symptoms will be neglected, making it difficult to diagnose neurotoxicity in these individuals. Finally, the majority of patients were treated with PES in conjunction with other drugs, and PES may not be solely responsible for the ultimate effectiveness. Further close monitoring of patients’ neurological symptoms, such as daily neurological examinations and electroencephalogram (EEG) monitoring, and analysis of *in vivo* and *in vitro* pharmacodynamics of different combination drug combinations may better assess the efficacy and safety of PES.

In conclusion, our study demonstrates that PES is an effective antimicrobial agent against MDR-GNB infections, particularly CRO, with little nephrotoxicity. In addition, the length of PES, aCCI, and p-PCT are key effectiveness predictors, and TDM and PK/PD investigations are required to establish the appropriate drug dosage exposure. In the absence of TDM, post-treatment PCT might be used as a reference indication. In fact, more multicenter clinical trials with large sample sizes are required to evaluate the efficacy and safety of PES. In the future, we will perform TDM in patients to conduct PD/PK studies to explore the optimal dose and regimen of PES for treating patients with CRO-induced infections. Further sample size expansion and multicenter studies will be conducted with feasibility.

## Data availability statement

The original contributions presented in this study are included in the article/supplementary material, further inquiries can be directed to the corresponding author.

## Ethics statement

The studies involving human participants were reviewed and approved by the Ethics Committee on Biomedical Research, West China Hospital of Sichuan University. Written informed consent for participation was not required for this study in accordance with the national legislation and the institutional requirements.

## Author contributions

XL: study design, data collection, data analysis, and original draft writing and editing. CZ, YL, HY, and JQ: consultation of antibacterial drugs and therapeutic use of polymyxin E sulfate. ZZ: consultation of antibacterial drug use and stewardship. XJL: study design, quality assessment, review of original draft and statistical analysis, supervision, and consultation of antibacterial drug use and stewardship. All authors contributed to the article and approved the submitted version.
